# Probiotics and Prebiotics: Role in Prevention of Nosocomial Sepsis in Preterm Infants

**DOI:** 10.1155/2013/874726

**Published:** 2013-01-14

**Authors:** Vrinda Nair, Amuchou S. Soraisham

**Affiliations:** ^1^Section of Neonatology, Department of Pediatrics, University of Calgary, Calgary, AB, Canada T1N 1N4; ^2^Alberta Children's Hospital Research Institute for Child and Maternal Health, University of Calgary, Calgary, AB, Canada; ^3^Department of Pediatrics, Foothills Medical Centre, Rm C211-1403-29th Street NW, Calgary, AB, Canada T2N 2T9

## Abstract

Nosocomial sepsis is associated with increased mortality and morbidity including neurodevelopmental impairment and prolonged hospital stay. Prevention of sepsis especially in the preterm infants in the neonatal intensive care unit remains a major challenge. The gastrointestinal tract is an important source of potential pathogens causing nosocomial sepsis as the immature intestinal epithelium can permit translocation of bacteria and yeast. The intestinal tract and its microflora play an important role in the immunity. Altering the gut microflora has been extensively studied for immunomodulation in preterm infants. Probiotics are live microorganisms which when administered in adequate amounts confer a health benefit on the host. Probiotics have been used for prevention and treatment of various medical conditions in children and adults. Studies on probiotics in premature infants have focused on normalizing intestinal flora, improvement in feeding intolerance, prevention of necrotizing enterocolitis and sepsis. In this paper, we discuss the intestinal bacterial colonization pattern; the rational for probiotics and prebiotic therapy with special focus on the prevention of nosocomial sepsis in preterm infants.

## 1. Introduction

Nosocomial infection (also referred to as late onset neonatal sepsis (LOS) or health care associated infection) in the neonatal intensive care units (NICU) is associated with morbidity and mortality, prolonged hospitalization, and increased medical costs [1]. Neonates, especially premature infants, are at high risk of acquiring nosocomial infections because of impaired host-defense mechanisms, limited amounts of protective endogenous flora on skin and mucosal surfaces at the time of birth, reduced barrier function of their skin, use of invasive procedures and devices, and frequent exposure to broad-spectrum antibiotic agents [1]. 

The nosocomial infection rate in the NICUs has increased over the past decades. About 6.2 to 33% of all neonates admitted to the NICU developed nosocomial infection [2]. Of all the very low birth weights (VLBW < 1500 gms) infants, 21% developed at least one episode of culture proven LOS [3]. The most common organisms causing nosocomial infection in neonates include *Staphylococcus*, *Escherichia coli, Klebsiella*, *and Candida*. *Coagulase negative staphylococcus* (CoNS) is responsible for almost half of the LOS [3, 4]. 

Neonatal sepsis has been associated with adverse neurological outcomes and poor growth in preterm infants [5–7]. There is an urgent need to identify appropriate strategies to prevent nosocomial infection. The most effective strategies for prevention of nosocomial infection include proper hand hygiene, prevention of central line-related blood stream infections, accurate diagnosis of infection, and limiting the use of unnecessary antibiotics [1, 8]. Borghesi et al. [9] have recently reviewed other strategies for the prevention of nosocomial infections including bovine lactoferrin, use of heparin for prevention of central line-related infection, judicious use of antimicrobial agents, and the use of specific antistaphylococcal immunoglobulins. Probiotics in the neonatal literature has generated a lot of debate in the last few years. In this paper, we examine the neonatal gut colonization, mechanisms of probiotics, and prebiotic and their role in prevention of nosocomial infections in preterm infants.

## 2. Bacterial Colonization of Developing Gut 

The fetus lives in a sterile intrauterine environment are protected by chorioamniotic membranes. At birth, the gastrointestinal tract is virtually sterile. The intestinal tract becomes colonized quickly after birth with a variety of ingested environmental and maternal flora [10]. The pattern and rates of neonatal colonization are influenced by gestational age, the route of delivery, maternal bacterial flora, antenatal and postnatal antibiotic use, hygiene of the neonatal environment, and type of feeding. There are significant differences in the intestinal colonization pattern between preterm and term infants. Healthy full term neonates delivered vaginally are colonized by anaerobic bacteria predominantly *Bacteroides* by one week of age. However, infants delivered by cesarean section exhibit delayed colonization by anaerobes and are colonized by *Klebsiella, Enterobacter, *and *Clostridia* [11]. Preterm infants' intestine tends to be colonized predominantly by *Bacteroides *spp., *E. coli,* and *Klebsiella. * The healthy commensal organisms like *Bifidobacterium *and *Lactobacilli* tend to appear only in the third week of life in preterm infants [12, 13]. Stools of breast fed infants have a predominance of *Bifidobacterium* and *Lactobacillus* species, which compete with *Bacteroides*, *Clostridia*, and *Enterobacteriaceae *found as intestinal flora in formula fed infants [10]. Exposure to maternal antibiotics as well as postnatal antibiotic therapy, total parenteral nutrition, or nursing in the incubator can delay or impair the intestinal colonization process [14].

The human intestinal tract continues to serve as host to a complex and dynamic society of nonpathological bacteria throughout life. The gastrointestinal tract is an important source of potential pathogens causing nosocomial sepsis as the immature intestinal epithelium can permit translocation of bacteria and yeast. Delayed enteral feeding, frequent use of antibiotic therapy, and altered acquisition of normal digestive microflora are important contributing factors for the increased risk of NEC in preterm infants and sepsis is often a complication of NEC. Normalizing the gut flora of preterm infants by administration of beneficial bacteria, in the form of probiotics, may help in reducing the incidence of NEC and nosocomial sepsis. Therefore, probiotics have been tried in preterm infants for this purpose. 

## 3. Probiotics

Probiotics are defined as live microorganisms which when administered in adequate amounts confer a health benefit on the host [15]. The term *probiotics *was initially used in the 1960s and comes from the Greek word meaning “for life.” Probiotics are commonly available as supplements (capsules, tablets, packets, or powders) and fermented dairy products such as yogurt. An ideal probiotic agent must be healthy, resist degradation by gastric acids and bile salts, adhere to intestinal epithelial cells, be considered nonpathogenic and non-invasive, modulate immune responses, be sensitive to usual antibiotics without the development of resistance, originate from microflora, and resist technological processing [16, 17].

The common microorganisms used as probiotics include

- Bacteria;
  - *Lactobacillus *species: *L. rhamnosus GG, L. acidophilus, L. caseii, L. plantarum, L. lactis, L. reuteri, and so forth; *
  - *Bifidobacterium *species:* B. bifidum, B. breve, B. infantis, B. lactis, and B. longum; *
  - *Streptococcus thermophiles;*
- Yeast:* Saccharomyces boulardii*.

## 4. Mechanism of Action of Probiotics

The exact mechanisms of how probiotics improve the health to the host are not clear. The effect of probiotics tends to be specific to a particular strain, so health benefit is not necessarily applicable to another strain, even within one species. The commonly used probiotics, *Lactobacillus *and *Bifidobacterium* species produce lactic acid, acetic acid, and propionic acid which lower the intestinal pH and suppress the growth of various pathogenic bacteria, thereby reestablishing the balance of the gut flora [18]. Probiotics may protect high-risk neonates and infants from developing sepsis and NEC by (i) increased barrier to migration of bacteria and their products across the mucosa, (ii) competitive exclusion of potential pathogens, (iii) modification of host response to microbial products [19], (iv) augmentation of IgA mucosal responses, (v) enhancement of enteral nutrition that inhibits the growth of pathogens and upregulation of immune responses [20, 21].

## 5. Evidence from Clinical Trials of Probiotics for Prevention of Sepsis and NEC in Preterm Infants

In children and adults, probiotics have been used for prevention and treatment of various medical conditions including acute infectious diarrhea, prevention of antibiotic-associated diarrhea, atopic disease, Crohn's disease, ulcerative colitis, and irritable bowel syndrome [22]. Studies on probiotics in premature infants have focused on normalizing intestinal flora, improvement in feeding intolerance, prevention of NEC, and sepsis. 

Millar et al. [23] reported one of the earliest studies of probiotics in neonates. Twenty preterm infants were randomized to receive either milk feeds or milk feeds *Lactobacillus GG* twice daily for two weeks from the day of initiation of feeds. There was no difference in sepsis and NEC between the two groups. Hoyos [24] reported a prospective cohort study with historical controls to examine the effectiveness of *Lactobacillus acidophilus* and *Bifidobacterium infantis *on reducing the incidence of NEC. A total of 1237 newborns (mean gestational age 35 weeks; mean birth weight 2040 g) during one year were treated with probiotics mixture. The primary outcome of NEC was reduced during the treatment year (3% versus 6.6%; *P* < 0.002) and NEC-associated mortality was reduced (37.8% versus 41.2%; *P* < 0.005). No difference was noted regarding nosocomial sepsis between the two groups (5.4% versus 5.5%). 

Table 1 describes the details of randomized control trials (RCT) on effects of probiotics on the neonatal outcome [25–40]. The primary outcome is NEC in majority of the trials and nosocomial sepsis is often a secondary outcome. We summarized the clinical trials on probiotics with nosocomial sepsis as one of the primary outcomes. 

Dani et al. [26] reported a double-blind RCT in 585 preterm VLBW infants to determine the effectiveness of *Lactobacillus GG* on urinary tract infection (UTI), bacterial sepsis, and NEC at 12 NICUs in Italy. No significant differences were observed between the groups: UTI (3.4% versus 5.2%), sepsis (4.7% versus 4.1%), and NEC (1.4% versus 2.8%). Awad et al. [35] examined the role of live and killed *Lactobacillus acidophilus* in reducing the incidence of nosocomial sepsis and NEC in 150 neonates (including 89 preterm infants). Infants who received either live or killed *Lactobacillus acidophilus* were less likely to develop nosocomial sepsis (45% versus 53.3% versus 63.3%), but it did not reach statistical significance. 

In an RCT by Mihatsch et al. [36], 183 VLBW infants <30 weeks of gestation were randomly assigned to have their milk feedings supplemented with *Bifidobacterium lactis *or placebo for the first 6 weeks of life. Primary outcome was the “incidence density” of nosocomial infections defined as periods of elevated C-reactive protein (>10 mg/L) from day 7 after initiation of milk feedings until the 42nd day of life (number of nosocomial infections/total number of patient days). There was no significant difference between the two groups with regard to the incidence density of nosocomial infections and the actual number of nosocomial sepsis. Reassuringly, none of the blood cultures grew *Bifidobacterium lactis.*

Recently, Romeo et al. [37] evaluated the role of probiotics for prevention of enteric Candida colonization and late onset sepsis in 249 preterm infants. The infants were randomized into three groups; one group supplemented with *Lactobacillus reuteri (LR)*, the second group supplemented with *Lactobacillus rhamnosus (LGG)*, and third group with no supplementation (control). The mean gestational age was 33 weeks. Candida stool colonization was significantly higher in control groups as compared with the probiotics groups. Only one infant in the LR group developed nosocomial sepsis; two infants in the LGG group developed nosocomial sepsis and nine infants in the control group developed nosocomial sepsis. 

## 6. Published Systematic Reviews/Meta-Analyses of Probiotics for Prevention of Sepsis and NEC in Preterm Infants

Four meta-analyses and two systematic reviewson probiotics in preterm infants have been published [41–46]. The details of the meta-analyses are shown in Table 2. Systematic review by Barclay et al. [41] included five RCTs using probiotics to prevent NEC in preterm infants. Meta-analysis was not performed because of the significant heterogeneity of the study design. The studies suggest that probiotics are likely to be useful in preventing and reducing the severity of NEC. Deshpande et al. [42] published the first meta-analysis involving 7 RCTs. This meta-analysis showed a lower risk of NEC ≥stage 2 (relative risk [RR] 0.36, 95% confidence interval [CI] 0.20–0.65, number needed to treat [NNT] 25, 95% CI 17–50) in the probiotic group compared to that in controls. There was no significant difference in the risk of sepsis (6 trials, *n* = 1355, RR 0.94, 0.74–1.20). Probiotic supplements reduce overall mortality (RR 0.47, 95% CI 0.30–0.73) but not mortality due to NEC and sepsis. Four additional studies are added in the updated meta-analysis by the same authors in 2010 [43]. The risk of NEC was significantly lower in probiotic group (RR 0.35, 95% CI 0.23–0.55). The risk of nosocomial sepsis was not reduced in probiotic group (RR 0.98, 95% CI 0.81–1.18). 

Cochrane review on probiotics included 16 trials randomizing 2842 infants [44]. The trials were highly variable with regard to enrolment criteria (i.e., birth weight and gestational age), baseline risk of NEC in the control groups, timing, dose, formulation of the probiotics, and feeding protocols. The meta-analysis showed that enteral probiotics supplementation significantly reduced the risk of severe NEC ≥stage 2 (RR 0.35, 95% CI 0.24–0.52) and all cause mortality (RR 0.40, 95% CI 0.27–0.60). There was no evidence of significant reduction of nosocomial sepsis (typical RR 0.90, 95% CI 0.76–1.07). The authors concluded that enteral supplementation of probiotics prevents severe NEC and all cause mortality in preterm infants. More studies are needed to assess the efficacy in extremely low birth weight infants (<1000 gms) and the most effective formulation and dose to be utilized.

Mihatsch et al. [45] reported a systematic review of the level of evidence for routine use of probiotics for reduction of mortality and prevention of NEC and sepsis in preterm infants. Fifteen trials were included; two of the trials were level of evidence-1b (LoE) and the remaining 13 were level 2b LoE. There was considerable heterogeneity among the studies. The authors reported that some probiotics are beneficial in relation to reduction of severe NEC (2b LoE) and reduction of mortality (2b LoE). Probiotics do not accelerate feeding advancement (1b and 2b LoE). There were no convincing benefits with regard to prevention of nosocomial sepsis. The authors conclude that there is insufficient evidence to recommend routine probiotics in preterm infants. However, there is encouraging data (2b LoE) which justifies the further investigation regarding the efficacy and safety of specific probiotics in circumstances of high local incidence of severe NEC.

The latest meta-analysis by Wang et al. [46] included 20 trials, including 4 studies published in Chinese biomedical literature. Probiotic supplement was associated with a significantly decreased risk of NEC in preterm VLBW infants (RR 0.33, 95% CI, 0.24–0.46). The risk of death was also significantly reduced in the probiotic group (RR 0.56, 95% CI, 0.43–0.73). There was no difference in the risk of sepsis between the probiotic group and placebo group (RR 0.90, 95% CI 0.71–1.15). The authors suggested the optimum type of probiotic supplement and the long-term effects need further study.

In summary, several meta-analyses have shown that enteral probiotics supplementation can reduce the risk of NEC and all cause mortality; however, there is no reduction in the nosocomial sepsis with probiotics supplementation in preterm infants. 

## 7. Limitations of Meta-Analyses on Probiotics in Preterm Infants

The various meta-analyses included RCTs with different inclusion criteria. Most of the RCTs included preterm infants <34 weeks [43, 46] and one included <37 weeks [44]. There are methodological differences among the RCTs included in the meta-analysis including different entry criteria for gestational age, the type and strain of probiotics used, and dosing and duration of intervention. Most of the meta-analyses included at least 10 different strains of probiotics. Most of the RCTs have NEC as primary outcome. None of the meta-analyses have extracted the data in relation to extremely preterm, extremely low birth weight, and those exclusively fed breast milk. 

## 8. Safety of Probiotics

Probiotics are regulated as dietary supplements and not subjected to the same rigorous standards as medications. A challenge with these products involves complexities of quality control with live microorganisms. Probiotics are generally considered safe and lack of serious adverse events from the multiple clinical trials in preterm infants is reassuring. However, there is always a danger of administering these supposedly nonpathogenic live organisms to the preterm infants whose immunity is already at stake for having been born premature. Several case reports of sepsis caused by probiotic organisms especially in immune-compromised individuals have been published [47–50].

Kunz et al. [47] reported two preterm infants with short gut who developed *Lactobacillus* sepsis while taking *Lactobacillus rhamnosus GG* supplements. Land et al. [48] reported LGG probiotic sepsis occurring in immunocompromised infants and children. Recently, Jenke et al. [50] published a case of *Bifidobacterium* spp. septicemia in a 600 gm infant who was being fed the probiotics (Infloran). There have also been reports of septicemia related to probiotic use in immunocompromised critically ill adult [51, 52]. *Lactobacillus *and *Bifidobacterium* are anaerobic microorganisms that require special culture techniques. Previous RCTs on probiotics did not use any special techniques for identifying *Bifidobacterias* and hence raises the question whether sepsis related to *Bifidobacterium* might be underestimated [50]. Therefore, increased awareness and implementation of appropriate culture techniques are important in future studies. 

Despite multiple studies on probiotics in preterm infants, the long-term effects and safety of probiotics in the preterm infants are still lacking. Oral probiotics given to preterm VLBW infants did not affect the growth and neurodevelopmental outcomes [53, 54]. Kalliomäki et al. [55] reported that probiotic supplements have a role in the prevention of atopic eczema in children up to 4 years of age. The Committee on Nutrition of the European Society of Pediatric Gastroenterology, Hepatology and Nutrition (ESPGAN) concluded that more studies are required to establish the safety and efficacy of probiotic and probiotic products in infants and children [56].

## 9. Prebiotics

Prebiotics are defined as “nondigestible food ingredients that beneficially affect the host by selectively stimulating the growth and/or activity of one or a limited number of bacterial species already established in the colon and thus in effect improve the host health” [57]. The prebiotics include oligosaccharides, glycoproteins, glycosaminoglycans, glycolipid, and mucin. Human breast milk oligosaccharides are a prototype of prebiotics, which have been shown to facilitate the growth of *Bifidobacteria *and* Lactobacilli* in the colon of breast fed infants [57]. Human milk oligosaccharides have been shown to be responsible for the development of immune system, the prevention of pathogenic infection, and, moreover, the modulation of infant gastrointestinal to bifidogenic microbiota [58, 59]. In addition to promoting the growth of friendly microbes, these substances also enhance the innate immunity, prevent the binding of pathogenic organism to the epithelium, and help in detoxifying the byproducts of these pathogens.

Srinivasjois et al. [60] in 2009 published a systematic review/meta-analysis on the efficacy and safety of prebiotic oligosaccharide supplementation of formula in reducing the incidence of NEC and sepsis in preterm infants. The authors included 4 trials on preterm infants <37 weeks (*n* = 126) who were on formula milk feeds [61–64]. The authors of 2 RCTs did not report data related to NEC or sepsis [61, 62]. The 2 authors reported that NEC did not occur in their study infants. There was no difference in the weight gain between prebiotics and control groups. The authors concluded that prebiotic-supplemented formula feeding increased stool colony counts of *Bifidobacterium* and *Lactobacilli* in preterm infants without adversely affecting the weight gain. 

In summary, there is no evidence of whether prebiotics have any role in reduction of nosocomial sepsis or NEC in preterm infants. 

## 10. Should We Start Routine ProbioticsSupplement for All VLBW Infants?

Several meta-analyses and systematic reviews have demonstrated that probiotics does not reduce the risk of nosocomial sepsis. However, there is evidence of reduction in the risk of NEC and all cause mortality with probiotic therapy in VLBW infants. There are important points to consider before staring routine probiotics. The different probiotic species have different effects and the optimal probiotic combination and dosing strategy are not clearly known. Probiotic preparations have not been rigorously regulated and some studies have shown inaccuracies in the reported organism species and the content and hence appropriate quality control are warranted. There have been reports of increased incidence of NEC with the routine use of probiotics in VLBW infants in Finland [65]. The ESPGHAN committee on nutrition concludes that there is not enough available evidence for the use of probiotics and prebiotics in preterm infants. 

## 11. Future Direction 

Currently one multicentre RCT investigating the effects of probiotics on LOS in VLBW infant (ProPrems trial) is underway in Australia and New Zealand [66]. The probiotic being used in this trial is a combination of *Bifidobacterium infantis*, *Bifidobacterium lactis*, and *Streptococcus thermophilus*. The primary outcome of the study is the incidence of LOS before 40 weeks or discharge home. Secondary outcomes include NEC, mortality, antibiotic usage, growth at 6 and 12 months, and atopic condition at 12 months corrected age. The result of this trial will help us in understanding of whether probiotic reduces the nosocomial sepsis in VLBW infants. Moreover, it is sufficiently power to demonstrate any significant adverse effects in infants below 1000 g birth weight and less than 28 weeks of gestation. 

## 12. Conclusion

Currently, there is no evidence regarding the usefulness of either probiotics or prebiotics for the prevention of nosocomial sepsis in preterm infants. There is evidence from clinical trials regarding the benefits of probiotics for prevention of NEC in preterm infants. The American Academy of Pediatrics and ESPGHAN committee suggested that there is no sufficient data at this time recommending routine probiotics for all infants. Results from multicentre trial powered to address this issue on safety and efficacy of probiotics are awaited. We hope that the ongoing multicentre trials may give better insight to the important aspect of whether probiotics prevents nosocomial sepsis in the extremely preterm infants. 

## Figures and Tables

**Table 1:** Clinical trials of probiotics for prevention of NEC and sepsis in neonates.

Study	GA (wk) BW (g)	Probiotic used	Dose and duration	Primary outcome	Comments
Kitajima et al. 1997, Japan [25]	<1500 *N* = 91	*BB*	0.5 × 10^9^ once daily from first feed for 28 days	Gut colonization by BB	No difference in sepsis
Dani et al. 2002, Italy [26]	<33 <1500 *N* = 585	*LGG*	6 × 10^9^ CFU once daily from first feeds till discharge	Urinary tract infection, bacterial sepsis, NEC	No difference in all three outcomes
Costalos et al. 2003, Greece [27]	28–32 *N* = 87	*SB*	10^9^/kg twice daily from first feed for 30 days	Gut function and stool colonization	No difference in sepsis
Lin et al. 2005, Taiwan [28]	<1500 *N* = 367	*LA, BI*	LA: 1004356 BI: 1015697 twice daily from day 7 until discharge	NEC	↓ NEC and sepsis rate in probiotic group (12.2% versus 19.3%)
Bin-Nun et al. 2005 Israel [29]	≤1500 *N* = 145	*BI, ST, BB*	BI: 0.35 × 10^9^ CFU ST: 0.35 × 10^9^ CFU BB: 0.35 × 10^9^ CFU once daily from first feed to 36 wks	NEC	↓ NEC in probiotic group. No difference in sepsis (43% versus 33%)
Manzoni et al. 2006, Italy [30]	<1500 *N* = 80	*LBC*	6 × 10^9^ CFU once daily from third day of life to 6 wks or discharge from NICU	Gut colonization by Candida	No difference in sepsis
Stratiki et al. 2007, Greece [31]	27–37 *N* = 78	*BL*	Preterm formula 2 × 10^7^ CFU/g started within 48 h.	Intestinal permeability	No difference in sepsis
Lin et al. 2008, Taiwan [32]	<34 <1500 *N* = 434	*BB, LA*	2 × 10^9^ CFU/day for 6 weeks	NEC or death	↓ NEC and mortality. ↑ sepsis risk in probiotic (19.8% versus 11.5%), but nonsignificant
Samanta et al. 2009, India [33]	<32 <1500 *N* = 186	*BI, BB, BL, LA*	2.5 × 10^9^ CFU/day till discharge	NEC, feed tolerance	↓ Sepsis in probiotic group (14.3% versus 29.5%)
Rougé et al. 2009 France [34]	<32 <1500 *N* = 94	*BL, LGG*	1 × 10^8^ CFU per day until discharge	Enteral feed intake at day 14	No difference in sepsis (33.3% versus 26.5%)
Awad et al. 2010, Egypt [35]	All neonate *N* = 150	*LA* (live and killed)	6 × 10^9^ CFU twice daily from day 1 till discharged	Sepsis and NEC	↓ sepsis rate in probiotic groups
Mihatsch et al. 2010, Germany [36]	<30 and <1500 *N* = 183	*BL*	12 × 10^9^ CFU/Kg/day for 6 weeks	Incidence density of nosocomial infection	No difference in sepsis
Romeo et al. 2011, Italy [37]	<37 <2500 *N* = 249	*LR* *LGG*	LR: 1 × 10^8^ CFU daily LGG: 6 × 10^9^ CFU daily from first 72 hrs to 6 wks or until discharge	Gut fungal colonization and late onset sepsis	Probiotics effective in prevention of gut colonization by Candida. No difference in sepsis
Braga et al. 2011, Brazil [38]	<1500 *N* = 231	*LC, BBr*	3.5 × 10^7^–3.5 × 10^9^ CFU Starting from day 2 till 30 days of life	NEC	No difference in sepsis (33.6% versus 37.5%)
Sari et al. 2011, Turkey [39]	<33 <1500 *N* = 221	*LS*	3.5 × 10^9^ till discharged	NEC, and mortality	No difference in sepsis (26.4% versus 23.4%)
Fernández-Carrocera et al. 2013, Mexico [40]	<1500 *N* = 150	*LA, LGG,* *LC, LP,* *BI, ST*	Multispecies probiotics 1 g/day	NEC	No difference in NEC and sepsis rate (56% versus 58.7%)

*BB: Bifidobacterium bifidus; BL: Bifidobacteruim lactis; LB: Bifidobacterium breve; LGG: Lactobacillus rhamnosus GG; LS: Lactobacillus sporogenes; SB: Saccharomyces boulardii; BBr: Bifidobacteria breve; BLo: Bifidobacterium longum; LC: Lactobacillus casei;* NEC: necrotizing enterocolitis; *ST: Streptococcus thermophillus; BI: Bifidobacterium infantis;* CFU: colony forming units; *LP: Lactobacillus plantarum; LR: Lactobacillus reuteri.*

**Table 2:** Meta-analyses of probiotics in neonates.

	Number of trials	Inclusion criteria	Number of infants	Sepsis (RR; 95% CI)	NEC (RR; 95% CI)	Mortality (RR; 95% CI)
Deshpande et al., 2007 [42]	7	<33 wks <1500 g	1393	0.94; 0.74–1.20	0.36; 0.20–0.65	0.47; 0.30–0.73
Deshpande et al., 2010 [43]	11	<34 wks <1500 g	2176	0.98; 0.81–1.18	0.35; 0.23–0.55	0.42; 0.29–0.62
Alfaleh et al., 2011 [44]	16	<37 wks <2500 g	2842	0.90, 0.76–1.07	0.35, 0.24–0.52	0.40, 0.27–0.60
Wang et al., 2012 [46]	20	<34 wks <1500 g	3816	0.90; 0.71–1.15	0.33; 0.24–0.46	0.56; 0.43–0.73
